# The role of tumor-associated macrophages and soluble mediators in pulmonary metastatic melanoma

**DOI:** 10.3389/fimmu.2022.1000927

**Published:** 2022-09-05

**Authors:** Kaifen Xiong, Min Qi, Tobias Stoeger, Jianglin Zhang, Shanze Chen

**Affiliations:** ^1^ The Department of Respiratory Diseases and Critic Care Unit, Shenzhen Institute of Respiratory Disease, Shenzhen Key Laboratory of Respiratory Disease, Shenzhen People’s Hospital (The Second Clinical Medical College), Jinan University, Guangdong, China; ^2^ The First Affiliated Hospital, Southern University of Science and Technology, Shenzhen, China; ^3^ Department of Dermatology, Xiangya Hospital of Central South University, Changsha, China; ^4^ Department of Plastic Surgery, Xiangya Hospital, Central South University, Changsha, China; ^5^ Institute of Lung Health and Immunity (LHI), Comprehensive Pneumology Center (CPC), Helmholtz Munich, Member of the German Center for Lung Research (DZL), Munich, Germany; ^6^ Department of Dermatology, Shenzhen People’s Hospital, The Second Clinical Medical College, Jinan University, Guangdong, China; ^7^ Candidate Branch of National Clinical Research Center for Skin Diseases, Shenzhen, China

**Keywords:** tumor-associated macrophages (TAMs), pulmonary metastatic melanoma, targeted therapy, soluble mediators, melanoma

## Abstract

Skin malignant melanoma is a highly aggressive skin tumor, which is also a major cause of skin cancer-related mortality. It can spread from a relatively small primary tumor and metastasize to multiple locations, including lymph nodes, lungs, liver, bone, and brain. What’s more metastatic melanoma is the main cause of its high mortality. Among all organs, the lung is one of the most common distant metastatic sites of melanoma, and the mortality rate of melanoma lung metastasis is also very high. Elucidating the mechanisms involved in the pulmonary metastasis of cutaneous melanoma will not only help to provide possible explanations for its etiology and progression but may also help to provide potential new therapeutic targets for its treatment. Increasing evidence suggests that tumor-associated macrophages (TAMs) play an important regulatory role in the migration and metastasis of various malignant tumors. Tumor-targeted therapy, targeting tumor-associated macrophages is thus attracting attention, particularly for advanced tumors and metastatic tumors. However, the relevant role of tumor-associated macrophages in cutaneous melanoma lung metastasis is still unclear. This review will present an overview of the origin, classification, polarization, recruitment, regulation and targeting treatment of tumor-associated macrophages, as well as the soluble mediators involved in these processes and a summary of their possible role in lung metastasis from cutaneous malignant melanoma. This review particularly aims to provide insight into mechanisms and potential therapeutic targets to readers, interested in pulmonary metastasis melanoma.

## Introduction

Cutaneous melanoma is a fatal skin malignant tumor derived from skin melanocytes. It is highly aggressive and is a major cause of skin cancer-related mortality; most patients have metastases the first time coming into the hospital (1, 2). The typical characteristics of cutaneous melanoma are early dissemination and subsequent metastatic colonization of multiple organs, for instance, lymph nodes, lung, liver, bone, and brain are common metastatic sites of skin melanoma (3). It’s worth noting that metastatic melanoma is the main cause of its high mortality and among multiple organs that may be colonized by cutaneous melanoma, the lung is one of the most common distant metastatic sites of melanoma (3–6). The incidence of pulmonary metastasis of melanoma accounts for 5% of all malignant metastases, and the mortality rate of pulmonary metastasis of melanoma is also very high (7). Primary melanoma is another condition of pulmonary melanoma, but the incidence of primary melanoma is only 0.01%, and its pathophysiology and therapeutic intervention are still unclear (7, 8). This review focuses on pulmonary metastatic melanoma, which accounts for the majority of cases of pulmonary melanoma. Hoping this review could provide some possible explanations for the relationship between melanoma lung metastases and TAMs, which may not only help provide possible explanations for cutaneous melanoma lung metastases but also provide potential new therapeutic targets for the treatment of melanoma lung metastases.

Tumor metastasis is not only driven by the intrinsic changes of tumor cells, the implicated cross-talk between tumor cells and their altered microenvironmental components all also play an important role in tumor metastasis. In addition, tumor cell metastasis is usually a stepwise cascading process, which mainly includes (1) tumor cell invasion in the primary site, (2) tumor cells intravasated into the vasculature, (3) tumor cell survival in the circulations, (4) tumor cells extravasated out of the vasculature, (5) tumor cells adapt, colonize and grow at the metastatic site (9, 10). The tumor microenvironment (TME) is considered to have a strong influence on tumorigenesis and the occurrence of tumor metastasis. The macrophages that accumulate around the TME are usually called tumor-associated macrophages(TAMs) (11). A large number of studies have shown that the TEM significantly promotes tumor metastasis usually caused by the increasing infiltration of TAMs, and TAMs are almost involved in the five cascade steps of tumor metastasis (12, 13). Recent studies have found that TAMs play an important role in tumor metastasis. For example, Prune-1 enhances the M2 polarization of TAMs in triple-negative breast cancer (TNBC) and promotes TNBC lung metastasis (14). In the development, metastasis, and invasion of ovarian cancer, programmed death-ligand 1 (PD-L1), an immunomodulation point factor of tumor-associated macrophages, plays an important role (15). Moreover, a study suggests that genetic variants in TAMs can predict clinical outcomes of bevacizumab therapy in patients with metastatic colorectal cancer (16). The elevated number of TAM in the melanoma TEM is often correlated with its poor prognosis, and TAMs targeted drugs have several clinical trials in the targeted therapy of melanoma, breast cancer, pancreatic cancer, prostate cancer, and other tumors (17–19). However, there is no definite hypothesis on the role of TAMs in the mechanism and targeted therapy of melanoma pulmonary metastasis at present.

This article will review the origin, classification, polarization, recruitment, regulation and targeting of TAMs, as well as the soluble mediators involved in these processes, and summarize their possible role in pulmonary metastases from cutaneous malignant melanoma. This review shall provide some possible mechanistic explanations and potential targeted therapy options for TAMs in pulmonary metastasis melanoma for readers.

## TAMs definition, origin, classification, and recruitment

### TAMs definition

Macrophages are a highly heterogeneous class of immune cells that are found in almost all tissues. Macrophages clustered around the TEM are called tumor-associated macrophages (TAMs) and are the most common immune cells in the TEM (20, 21). A large number of studies have shown that the increased accumulation of TAMs in tumor tissue is related to the poor prognosis of tumor patients (15, 22). With increasing the infiltration of TAMs, the tumor cell proliferation, and invasion could be promoted and angiogenesis could be stimulated. Moreover, TAMs can exert anti-tumor immune responses by mediating the production of growth factors, chemokines, and other immune components by T cells, thus regulating tumor metastasis (15, 22–25).

### TAMs origin

Blood monocytes derived from bone marrow hematopoietic stem cells were previously considered the generic origin of macrophages (20, 26). However, recent studies revealed that tissue macrophages of various organs such as the lung, brain, and liver embryonically originate from yolk sac progenitor cells (27). Consistent with this, there are two possible explanations for the origin of TAMs (1) tissue-resident TAMs: yolk sac progenitor-derived macrophages in various tissues may alter their phenotype or activation during tumorigenesis status; (2) tumor-induced TAMs (Monocyte-derived TAMs): Monocytes themselves undergo a distinct differentiation stage during tumor growth and eventually become macrophages (26, 28). In different stages of tumorigenesis and development, the source of dominant macrophages may be different. Tissue-resident TAMs may predominate in the early stages of tumor growth, while tumor-induced TAMs may predominate in the later stages of tumor growth. About 30% of tumor cells in melanoma are TAMs mainly derived from circulating monocytes, which are considered to be the most abundant leukocytes in melanoma lesions (29).

### TAMs classification

Macrophages are generally classified according to their function and their response to polarizing agents. Naive/unpolarized macrophages are called M0, and polarized macrophages are generally divided into M1 (classically activated macrophages) and M2 (alternatively activated macrophages), M2 macrophages can be further divided into Ma, Mb, Mc, Md subtypes (30, 31). Although, with the deepening of the understanding of macrophage polarization conditions, numerous new macrophage phenotypes, for example, Christian A. Gleissner, et al. identified M4 macrophages by comparing the transcriptome of M-CSF (macrophage colony-stimulation factor) and CXCL (4CXC Chemokine Ligand 4) induced macrophages *in vitro*, providing a novel starting point for atherosclerosis and other disease-related research; macrophages defined as M17 for secreting IL17 are also new macrophage phenotypes that have been recently identified (32, 33). The continued discovery of new macrophage phenotypes amply demonstrates the heterogeneity and plasticity of macrophages; however, the commonly used classification remains M1-M2, which differ in metabolic characteristics, markers, and gene expression profiles (31). M1 macrophages are involved in the anti-infective response together with helper T1 cells (Th1). It exerts its pro-inflammatory and anti-tumor effects by secreting pro-inflammatory cytokines such as interferon (IFN)-γ, IL-6, IL-12, tumor necrosis factor (TNF)-α, lipopolysaccharide (LPS) or molecules that regulate inflammation, and by producing substances such as nitric oxide synthase (NOS, a synthase involved in arginine metabolism) and reactive oxygen species (ROS) (15, 34). While the characteristics of M2 macrophages tend to favor tissue repair and tumor progression. M2 macrophages exert anti-inflammatory effects by secreting anti-inflammatory cytokines, such as IL-4, IL-10, IL-13, and TGF-β, and producing scavenger receptors and other substances; promoting proliferation, invasion and metastasis of tumor cells by inhibiting T-cell activity (15, 35, 36). The biological process of conversion between M1 (anti-tumor) and M2 (pro-tumor) in response to microenvironmental signals is termed “macrophage polarization” (35). Studies have shown that TAMs exist either two phenotypes on the tumor, for example, a study revealed that malignant melanoma TAMs as a heterogeneous phenotype with both M1 and M2 markers (37); However, the term “TAMs” now mainly refers to M2 macrophages because the functional characteristics of TAMs are more similar to M2 macrophages rather than to M1 macrophages (38).

### TAMs recruitment

Expansion of tissue-resident TAMs and/or increased recruitment of monocyte-derived TAMs are important features of cancer. Cytokines, chemokines, growth factors, and other signals from tumor cells and stromal cells can induce the recruitment of TAMs to tumor sites (22, 39, 40). Several studies have found high levels of accumulation of tissue-resident macrophages and monocyte-derived macrophages in tumors such as glioblastoma, hepatocellular carcinoma and lung cancers (41–43). We therefore pondered whether the recruitment of TAMs also plays an important role in melanoma lung metastasis?

Colony-stimulating factor 1 (CSF-1), also known as macrophage colony-stimulating factor (M-CSF), is a secreted cytokine that differentiates hematopoietic stem cells into monocytes, macrophages, or other related cell types (44). CSF-1 acts as a major lineage regulator of macrophages and is also a recognized recruiter of macrophages. CSF-1 affects macrophages and monocytes through increased phagocytic, chemotactic activity and tumor cytotoxicity or other ways to affect macrophages and monocytes (44). Extensive overexpression of CSF-1 can be observed at the invasive edge of the various tumor, and it is involved in the polarization of M2 macrophages by interacting with its ligand CSF-1R; Its overexpression is also associated with a significant increase in metastasis (22, 45). Depletion of CSF-1/CSF-1R was shown to significantly inhibit tumour progression and metastasis in various tumour models (22, 44, 46). On the contrary, restoration of CSF-1 expression can accelerate tumor progression and metastasis in a CSF-1 mutant mouse xenograft model (46).

CCL2 is another well-established macrophage recruiter and M2 stimulator in addition to CSF-1. It is reported that CCL2 could induce the differentiation of macrophage into protumor phenotypes through CC chemokine receptor 2 (CCR2); In mouse tumor models such as colorectal cancer, prostate cancer, and melanoma, CCL2 recruit TAMs to tumor sites through ligand-receptor interactions with CCR2 or other forms of action (47–50). When the CCL2/CCR2 interaction was blocked, the metastatic dissemination of tumors in the mouse was significantly inhibited, the survival of the mouse was prolonged and the expression levels of tumor-promoting cytokines were reduced (47, 51). In a study by Xiaojing Chen, Yuanrun Deng et al. CCL8 act as another ligand for CCR2 was found to be highly overexpressed in human cervical cancer, which involved in the recruitment of TAMs in hypoxic regions (52). Moreover, the high expression of CCL2 in tumors such as esophageal carcinogenesis and clear cell renal cell carcinoma is associated with increased TAMs infiltration and metastatic events (53, 54).

It is well known that hypoxia promotes malignant tumor behavior through multiple mechanisms, including promoting glycolysis, antagonizing apoptosis, inducing immune escape, and inducing drug resistance, and is a common feature of most solid tumors (55–57). Studies have shown that hypoxia has an important impact on the recruitment of TAMs and that hypoxia captures disseminated macrophages by downregulating chemokine receptors expressed on macrophages (38). In addition, TAMs are recruited to the hypoxic TME through CCL2, VEGF-A, endothelin-2, semaphorin 3A (SEMA3A), stromal cell-derived factor 1α (SDF1α), and oncostatin M, etc. (38, 58, 59). TAMs are involved in angiogenesis and tumor spread through upregulation of hypoxia-inducible factor (HIF)-1a and HIF-2a when they are recruited to the hypoxic environment (38). The recruitment of TAMs by hypoxia facilitates the conversion of the TME into more hospitable sites for a tumor cell.

**Figure 1:**
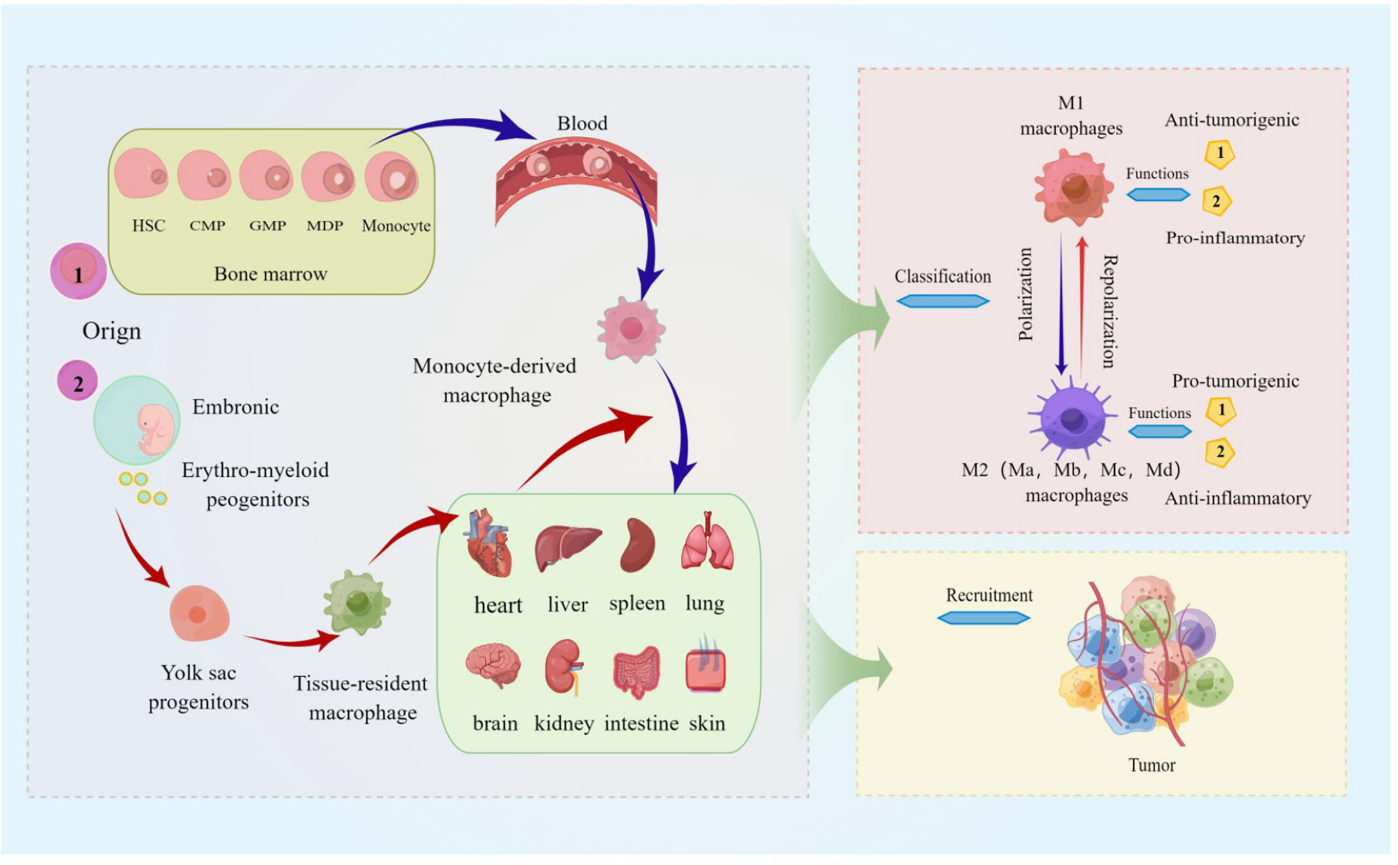
The source, function, classification, and recruitment of TAMs. The picture displayed that TAMs are derived from blood mononuclear cells from bone marrow hematopoietic stem cells, called monocyte-derived TAMs (or tumor-induced TAMs) and yolk sac progenitors, and reside in organs such as the lung, brain, liver, and skin. Macrophages polarized in tumors are divided into M1 (classically activated macrophages) and M2 (alternatively activated macrophages), and M2 macrophages can be further divided into Ma, Mb, Mc, and Md subtypes. M1 macrophages are characterized by the secretion of pro-inflammatory cytokines involved in anti-infective responses, and the immunosuppressive phenotype of M2 macrophages tends toward tissue repair and tumor progression; TAMs are activated by cytokines, growth factors, etc. can be recruited to tumor sites.

Besides, ligand-receptor interactions such as CCL3/CCR1, CCL3/CCR5, CCL5/CCR5, CX3CL1/CX3CR1, and VEGF-A/VEGFR1 and chemokines such as CCL7, CCL8, CCL9, CCL18, CCL20, IL-13…all can contribute to the survival, progression, and metastasis of various tumor including melanoma by recruiting TAMs to TEM (40). For example, the chemokine CCL20 is recognized by the CCR6 ligand expressed in melanoma and promotes melanoma growth and metastasis *in vivo (* 60). It is reasonable to speculate that the recruitment of TAMs has a potential role in pulmonary metastatic melanoma. We still further describe the source, function, classification, and recruitment of TAMs in detail, as shown in **Figure 1**, hoping to better help readers understand the relevant definitions of TAMs.

## The roles of TAMs in pulmonary metastatic melanoma

### Pulmonary metastatic melanoma

Invasion and metastasis are the main causes of tumor death, and it is reported that the mortality caused by malignant tumor metastasis accounts for more than 90% of cancer mortality (61). Metastasis generally refers to the migration and spread of cancer cells from the primary sites to surrounding and distant organs; most malignant solid tumors could metastasize from the primary organ to other organs, such as the lung, liver, brain, and bone. Such a metastatic process is a gradual process termed the “metastatic cascade” (62). Like most solid tumors, melanoma also metastasizes to other organs. Early lymph node metastasis is one of the typical features of melanoma, the lung is one of the most common distant metastases of melanoma, and it is reported that about 18% of melanoma patients developed lung metastases during follow-up (63, 64). Moreover, the clinical prognosis of melanoma lung metastases is poor, and the 1-year survival rate of patients with melanoma lung metastases has been reported to be only about 30%-60% (65).

**Figure 2:**
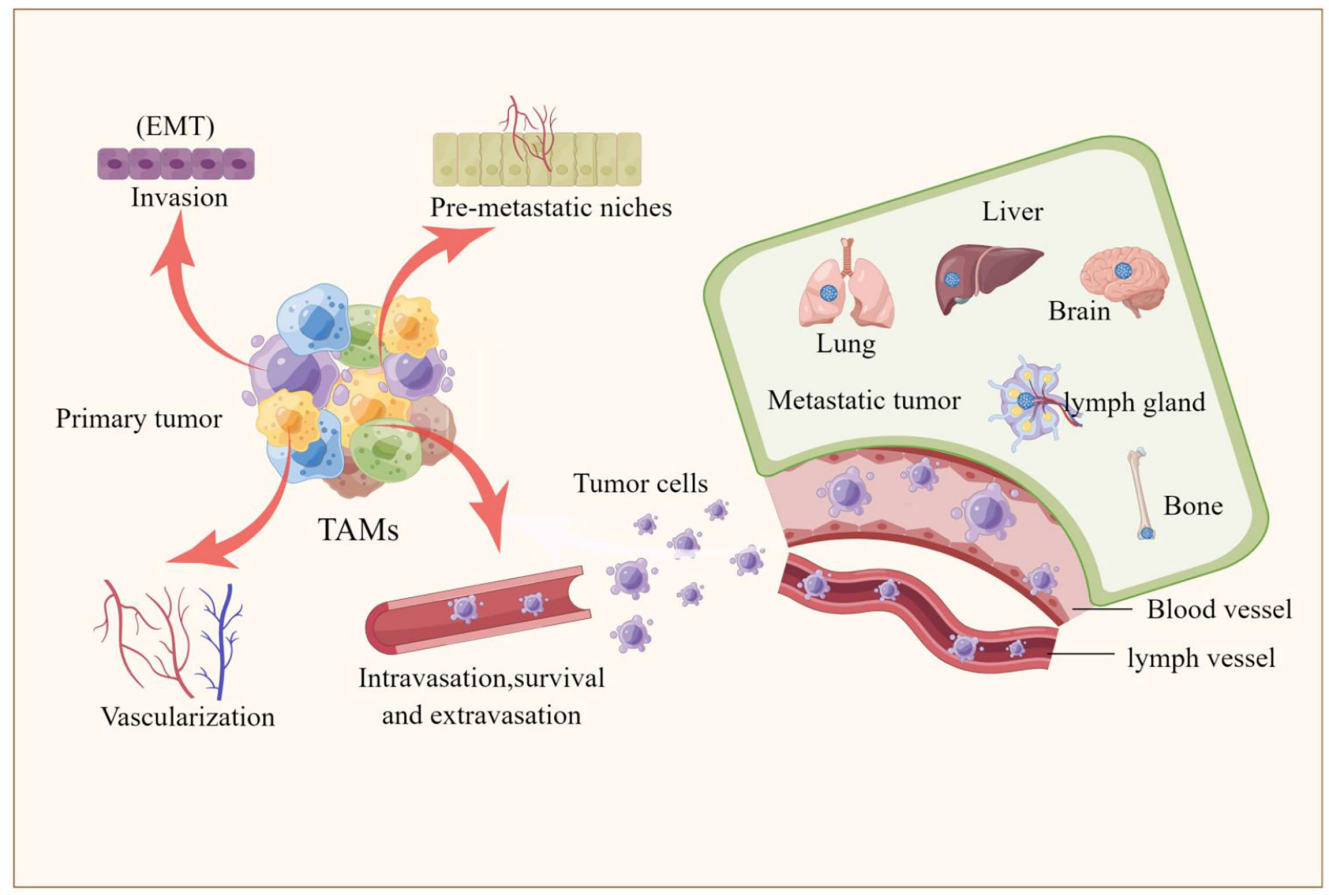
TAMs are involved tumor metastasis. The picture illustrated that TAMs are involved in every step of the metastatic cascade, including the formation of pre-metastatic ecological sites, invasion of tumor cells, survival of circulating tumor cells, extravasation and colonization of tumor cells, and the involvement of TAMs in regulating the epithelial-mesenchymal transition (EMT) process that promotes the formation of liver, brain, lung, bone and lymph node metastases.

Epithelial-mesenchymal transition (EMT) is the process by which epithelial cells acquire mesenchymal properties and is associated with multiple functions such as tumour progression, metastasis and drug resistance (66). Recently, a series of studies have shown that TAMs are involved not only almost in each step of the metastatic cascade, such as the formation of pre-metastatic ecological niches, infiltration of tumour cells, survival of circulating tumour cells, extravasation and colonization of tumour cells, but also in the regulation of the EMT process (12, 15, 67–69). TAMs exhibit many important biological functions from different aspects during tumor progression. Here, we mainly focus on the role of TAMs in the process of pulmonary metastasis of melanoma. TAMs are involved in almost every step of metastasis as described below, also shown in **Figure 2**.

### Pulmonary metastatic melanoma and TAMs

#### TAMs promote invasion of melanoma tumor cells

Invasion is generally considered to be the initiation of tumor metastasis. EMT and TAMs plays an important role in tumor invasion. On the one hand, EMT is a major event in the morphological transformation of tumor cells that acquire the invasive ability to escape from the border of the basement membrane to the surrounding mesenchyme, which contributes to tumor cells invasion and metastasis (70). On the other hand, compatibility factors secreted by TAMs, such as IL-8, TNF-α, and transforming growth factor-β (TGF-β), are involved in the regulation of the EMT process and promote metastasis (67, 71).

Abundant shreds of evidence indicate that the proportion of TAMs infiltrating melanoma is increased, and the high expression level of macrophage markers in melanoma tissue is closely related to poor prognosis (72, 73). A study suggests that high levels of reactive oxygen species (ROS) in primary melanoma may increase cytokine-tumor necrosis factor-α (TNF-α) secretion through MAPK/ERK kinase 1-mediated peroxisome proliferator-activated receptor γ (PPARγ) translocation to the nucleus, and it thus enhanced the invasion of TAMs in melanoma (37). These studies all provide favorable evidence to support the idea that TAMs promote invasion and cause melanoma lung metastasis.

#### TAMs promote vascularization of melanoma tumor cells

The process of forming new blood vessels from existing blood vessels is called angiogenesis. Angiogenesis is a vital condition for tumor cell proliferation and generation (74). Many tumors, including melanoma, metastasize through the vascular system and/or the lymphatic system. Existing evidence strongly supports that TAMs drive tumor angiogenesis through vascular endothelial growth factor (VEGF) matrix metalloproteinase (MMP9); TAMs stimulate the remodeling of established vasculature into a more tortuous and leaky form, which is beneficial to tumors dissemination of cells (15, 75, 76). When TAMs are absent, vessel density can be reduced by approximately 40% (76). In addition, studies have shown that pro-angiogenic molecules such as nitric oxide (iNOS), MMP7, CXCL8, IL1, and fibroblast growth factor (FGF)-2 can regulate tumor angiogenesis through TAMs (75, 77).

Studies show that the process of VEGF-C (tumor overexpressed ligand)/VEGFR-3 (receptor for VEGF-C expressed on TAMs) promotes lymphangiogenesis by directly affecting the activity of lymphatic endothelial cells (lymphatic endothelial cells) or indirectly increasing the secretion of ductal proteins to promote lymphangiogenesis, which supports the hypothesis that lymphangiogenesis is closely related to TAMs and also further suggests that TAMs play an important role in tumor invasion and metastasis (78, 79). The research carried out by Peiwen Chen et al.shows that TAMs promote angiogenesis and melanoma growth through paracrine and autocrine adrenomedullin. When adrenomedullin levels are reduced or secretion is inhibited, angiogenesis and melanoma growth *in vivo* are correspondingly inhibited (80). Monocyte chemoattractant protein-1 (MCP-1) is a member of the CC-motif chemokine family (as CCL2); MCP-1, a chemokine, is also one of the key agonists in the recruitment of macrophages to tumor sites (81). Both *in vivo* and *in vitro* studies have demonstrated that MCP-1 triggers a rich vascular network through M2 macrophages, and targeting TAMs to inhibit MCP-1 reduces angiogenesis and tumor growth in human melanoma xenografts (47).

Taken together, TAMs play an important role in regulating tumor angiogenesis and promote the vascularization of tumor cells (including melanoma cells)through different pathways, which may provide some explanation for melanoma lung metastasis.

#### TAMs promote the intravasation, survival in the circulation, and extravasation of melanoma tumor cells

Intravasation is defined as the process by which tumor cells leave the primary tumor and enter the circulation (generally into the blood circulation); tumor cells that enter the circulation survive and participate in the circulation and then leave the circulatory system, and the process of entering the secondary site from the primary tumor is called extravasation (82). Intravasation, survival, and extravasation of tumor cells in the circulatory system are all important steps in the cascade of events leading to tumor metastasis. Multiphoton *in vivo* imaging experiments showed that macrophages were mainly concentrated in the periphery of the tumor, and the density of macrophages in the center of the tumor decreased; the macrophages in the tumor were localized to the blood vessels and assisted the intravasation of tumor cells (83). Another study found that the intravasation of tumor cells was always accompanied by macrophages within one cell diameter (84). These studies provide strong evidence that TAMs enhance the ability of cancer cells to invade adjacent normal tissue.

Clinical observations suggest that the tumor metastasis microenvironment (TMEM) is composed of tumor cells, TAMs, and endothelial cells (15). In TMEM, TAMs can produce EGF (epidermal growth factor) and secrete chemokine CCL18, and tumor cells can also secrete chemokine CCL18 and produce cytokine CSF-1, all of which play important roles in the process of tumor cell invasion. For example, a study by Jingqi Chen et al. found that CCL18 secreted by TAMs in breast cancer induces integrin accumulation in cancer cells, which further promotes the adhesion of integrin-aggregated cancer cells to the extracellular matrix and then intravasation (85). The chemokine CCL18 produced by melanocytes itself can be increased and released under the induction of long non-coding RNA (LncRNA) CRNDE, and promote the proliferation, invasion, and metastasis of melanoma (86).

Tumor cells, which intravasated the circulation, also need to survive and circulate in the circulatory system. On the one hand, tumor cells evade recognition and killing by cytotoxic immune cells by clustering with platelets in the circulatory system; On the other hand, the survival of many tumor cells is protected by chemokines or cytokines secreted by macrophages (87, 88). Regarding the involvement of TAMs in tumor cell extravasation and tumor cell colonization, existing evidence supports that when tumor cells interact, macrophages have a higher extravasation rate. Therefore, the number of tumor cells that occurs extravasation was significantly reduced when macrophages are depleted (89).

Are TAMs also involved in promoting the endocytosis, circulating survival and extravasation of melanoma cells during pulmonary metastasis of melanoma? Based on the results of the above-mentioned studies, we believe that the answer is more than likely also in the affirmative.

#### TAMs prepare sites for melanoma tumor cells: pre-metastatic niches

The favorable microenvironment created by the primary tumour for secondary organs and/or tissues to which it subsequently metastasizes (metastatic target organs or tissues) is known as the pre-metastatic niche (PMN). the PMN is initiated and established through a complex interaction between growth factors, inflammatory factors, chemokines, bone marrow-derived cells and local stromal components in the primary tumour (90). Studies have shown that macrophages are one of the key determinants of PMN formation. Tissue-resident TAMs, such as osteoclasts and alveolar macrophages, are involved in PMN formation (91, 92). What’re more, soluble mediators such as CSF-1, VEGF, TGF-α, and exosomes and tissue inhibitors of metallopeptidase (TIMP) that are involved in the recruitment of TAMs can also mobilize TAMs to aggregate in PMNs (92).

TAMs interact with dendritic cells(DC) and T helper cells (Th1 cells), impairing the antigen presentation and antitumor behavior of these immune cells; TAMs regulate tumor angiogenesis and extravasation through matrix-derived factors (SDFs) and matrix metalloproteinases, etc., which in turn promote the formation of PMNs from tumor cells (CTCs) (15, 92, 93). Considering the preferential metastasis of lung-homing melanoma cells in melanoma metastasis and the important regulatory role of PNM in tumor metastasis, it is reasonable to speculate that TAMs and soluble mediators play an important role in melanoma lung metastasis by promoting PMN formation.

## TAMs regulation and pulmonary metastasis melanoma

**Figure 3:**
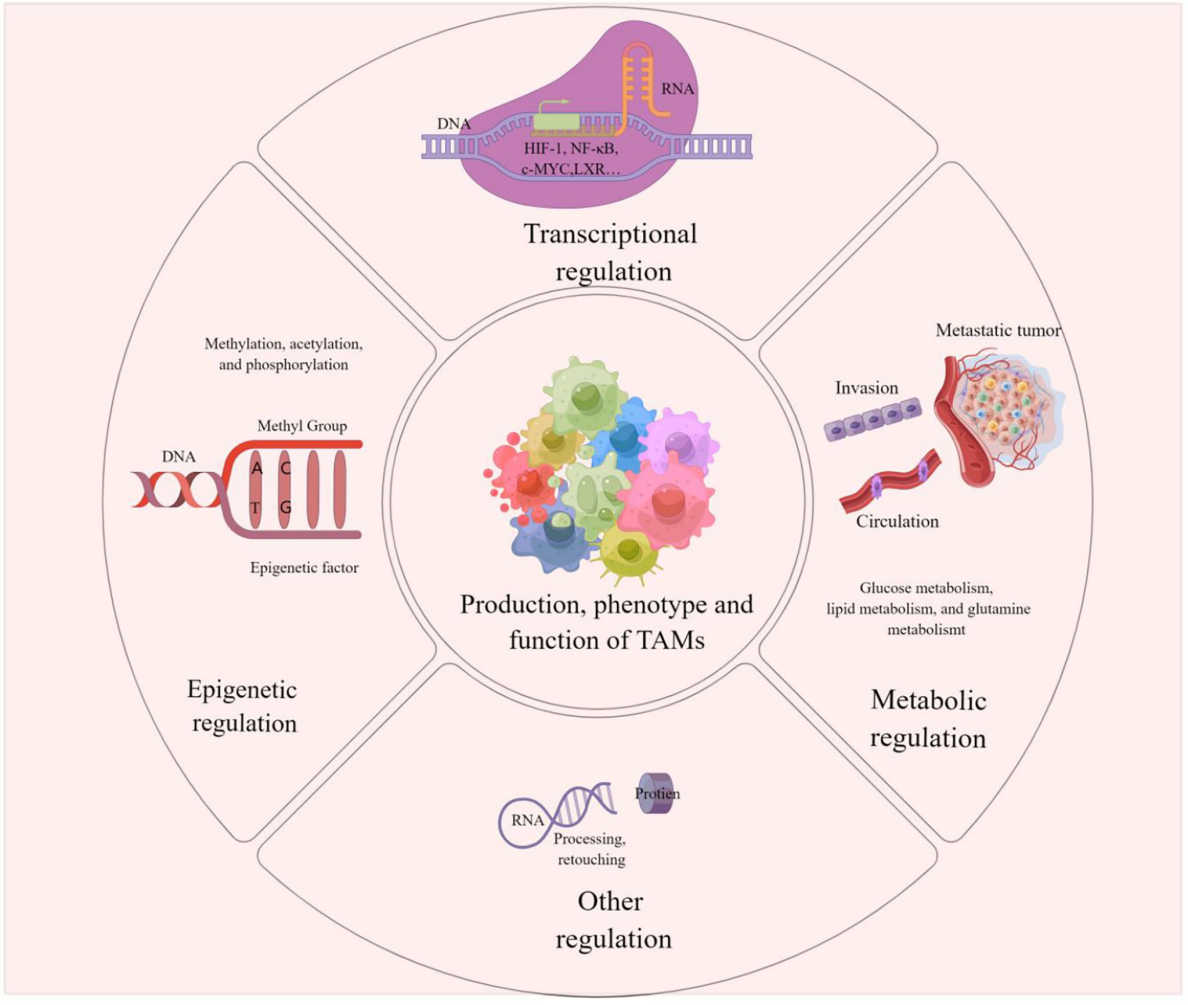
TAMs regulation This picture demonstrates that the production, phenotype and function of TAMs are regulated in epigenetic, transcriptional, metabolic or other different ways. These modulations influence tumorigenesis, metastasis and drug resistance on Pulmonary metastases in melanoma can also be influenced by the regulation of TAMs.

It has been reported that affecting TAM regulation at the transcriptional, epigenetic and metabolic levels could be an effective cancer treatment modality. The regulation of TAMs can influence its production, phenotype, and function, which in turn affect tumor development and metastasis, and drug resistance. The clinical application of corresponding activators or inhibitors for different regulatory modalities and related pathways is expected to target TAMs to improve the efficacy of tumor immunotherapy. We present the modalities of TAMs regulation in **Figure 3**.

We speculate whether regulating the production, phenotype and function of TAMs influence pulmonary metastasis from melanoma. Accordingly, does the application of activators or inhibitors that modulate key factors also improve the efficacy of therapy for lung metastases from melanoma?

### Epigenetic regulation of TAMs and pulmonary metastasis melanoma

Epigenetics refers to developmental or environment-induced modifications that control the expression of information encoded in DNA in a tissue- and environment-specific manner without altering the genetic code (94). Various cellular functions, such as cell differentiation, cell activation, and transformation, are regulated by epigenetic changes in gene expression. Epigenetic dynamics of DNA methylation and histone modifications (such as methylation, acetylation, and phosphorylation) are associated with altered gene expression (95). The development, polarization, and activation of macrophages are also controlled by DNA and histone modifications. For example, DNA methylation and myeloid differentiation, Histone modifications in M1 macrophage activation, and M2 macrophage polarization by Jmjd3(an H3K27-specific demethylase) are currently clear mechanisms for the epigenetic regulation of macrophage phenotype and function (96). In addition to the epigenetic effects of DNA methylation and histone modification on the phenotype and function of TAMs, emerging data suggest that epigenetic changes in noncoding RNAs also have regulatory effects on TAMs. For example, many miRNAs involved in the production of IL-10 and the expression of PD-L1 in TAMs exert immunosuppressive effects in an epigenetic manner, helping to identify new therapeutic targets and providing a research reference for improving tumor sensitivity to immune responses (97).

It is noteworthy that changing epigenetic regulation in macrophages selectively targets M2 macrophages and removes tumor-promoting TAMs while retaining only tumor-suppressed M1 macrophages (98, 99). Pharmacological modulators of many epigenetic enzymes are currently in clinical use and can be used to treat tumors with high TAM infiltration (99). Much research has been done on the epigenetic enzymes of M1s and their regulators, but much less is known about the epigenetic regulation of M2s, especially about cancer (99).

Taken together, epigenetic regulation affects tumorigenesis, progression, and metastasis by affecting the phenotype and function of TAMs. Given that melanoma is also one of the tumors subject to epigenetic regulation, it is reasonable to speculate that epigenetic regulation in TAMs may be an important reference direction for studying the mechanism and treatment of pulmonary metastatic melanoma.

### Transcriptional regulation of TAMs and pulmonary metastasis melanoma

It is well known that malignant tumors usually have hypoxia, and the activation of two transcription factors, hypoxia-inducible factor-1 (HIF-1) and nuclear factor kappa b (NF-κB) is closely related to the occurrence of hypoxia. Circulating hypoxia affects the expression of angiogenesis-inducing cytokines such as VEGF-A, CCL2/MCP-1, etc., and recruits various cells into the tumor niche, where they are transformed into tumor-associated macrophages (TAM) involved in tumorigenesis, etc. Through the regulation of transcription factors HIF-1 and NF-κB and their related pathways, regulating the generation of TAMs may be one of the potential tumor-targeted therapeutic options (100). STAT-3 is also one of the key factors to initiate the transcriptional program of TAMs (101). In addition, research shows that transcription factor EB (TFEB) expression is significantly reduced in breast cancer. TFEB controls the phenotype and function of TAMs through multiple autophagy/lysosome-dependent and independent pathways, thereby promoting breast tumor development; Conversely, activation of TFEB is expected to be a target for TAMs for tumor immunotherapy strategies including breast cancer (102). The Liver X receptor (LXR) is one of the transcription factors in the nuclear receptor family that is activated by oxysterols and synthetic high-affinity agonists; A study shows that in a mouse model of lung cancer, pharmacological LXR activation can regulate TAM gene expression, thereby exerting an anti-tumor effect (103). In addition, studies show that the transcription factor c-Maf is a key controller of immunosuppressive macrophage polarization and function in cancer; Numerous M2 macrophage-related genes are controlled by c-Maf, which in turn promotes M2 macrophage-mediated T cell suppression and tumor progression. In a subcutaneous LLC tumor model, inhibition of c-Maf partially overcomes resistance to anti-PD-1 therapy; Likewise, c-Maf is expressed in human M2 and tumor-infiltrating macrophages/monocytes as well as circulating monocytes in human non-small cell lung cancer (NSCLC) patients and plays an important role in regulating its immunosuppressive activity (104).

Overall, various transcription factors including c-MYC, LXR, TFEB, etc., regulate the phenotype and function of TAMs in solid tumors such as lung cancer and breast cancer, thereby affecting tumor progression and metastasis. The clinical application of corresponding transcriptional activators or transcriptional inhibitors is expected to provide a new strategy for targeting TAMs in the treatment of tumors. Lung metastatic melanoma derived from melanoma has a close relationship with TAMs. The role of TAMs in pulmonary metastatic melanoma through transcriptional regulation needs more research, which not only contributes to providing a certain explanation for the development of pulmonary metastatic melanoma but may also provide possible strategies for targeting TAMs in the treatment of pulmonary metastatic melanoma.

### Metabolic regulation of TAMs and pulmonary metastasis melanoma

As an important immune cell in TME, TAMs are closely related to poor tumor prognosis, drug resistance, enhanced angiogenesis, and tumor metastasis; A complete interpretation of the pro-tumor and anti-tumor metabolic switches in TAMS is critical for understanding immune escape mechanisms in cancer (105). Currently, a few of researches have been done on the intertwined relationship between metabolism and macrophages in the context of cancer, and the interaction between the two is not fully defined. Considering that pulmonary metastatic melanoma is closely related to TAMs like many solid tumors, the study of the regulation of TAMs may provide some explanation for the mechanism of pulmonary metastatic melanoma and its therapeutic direction.

A tumor hypoxic environment induces transcription of genes related to glucose and nitrogen metabolism. Hypoxia of the TME increases the levels of arginase-1 and man-ose receptor (CD206) on TAMs, and TAMs present in hypoxic regions induce the expression of HIF-1α, which induces a switch to glycolytic fermentation (106). Furthermore, a study shows that hypoxic TAMS strongly upregulated the expression of Redd1 (a TOR complex 1-MTORC1 inhibitor), a negative regulator of the mechanistic target of rapamycin (mTOR, a key nutrient, and energy sensor); Metabolic changes promote tumor angiogenesis and metastasis by inhibiting the glycolysis of hypoxic TAMs, inhibiting their angiogenesis and immunosuppressive effects (107). Moreover, exosomes derived from tumor cells can affect the differentiation of macrophages by altering the miRNA profiles of TAMs. For example, in the study of ovarian cancer, it was found that hypoxia induced the expression of miR-940 in tumor exosomes and stimulated macrophages polarized toward the M2 phenotype; This also suggests that the metabolic program in TAMs is a combination of hypoxia and cytokines in the microenvironment (108, 109). Glucose metabolism, lipid metabolism, and glutamine metabolism all play an important role in the regulation of TAMs metabolism, and therapeutics targeting metabolic pathways in TAMs is also one of the alternative strategies for cancer treatment (105).

In general, some studies have shown that metabolic alterations of TAMs in a variety of solid tumors promote or inhibit their progression and metastasis. The metabolic effects on substances such as sugar and liposomes in melanoma TEM may also cause alterations in TAMs, which may affect melanoma lung metastasis. How alterations in the metabolism of TAMs affect their phenotype and function, and their impact on tumor growth and metastasis, including melanoma, remains to be revealed by more studies. The role of metabolic regulation of TAMs in pulmonary metastatic melanoma is also worthy of further investigation, as this may provide possible mechanistic explanations and even potential therapeutic targets for pulmonary metastatic melanoma.

## TAMs targeted treatment strategies in pulmonary metastatic melanoma

**Figure 4:**
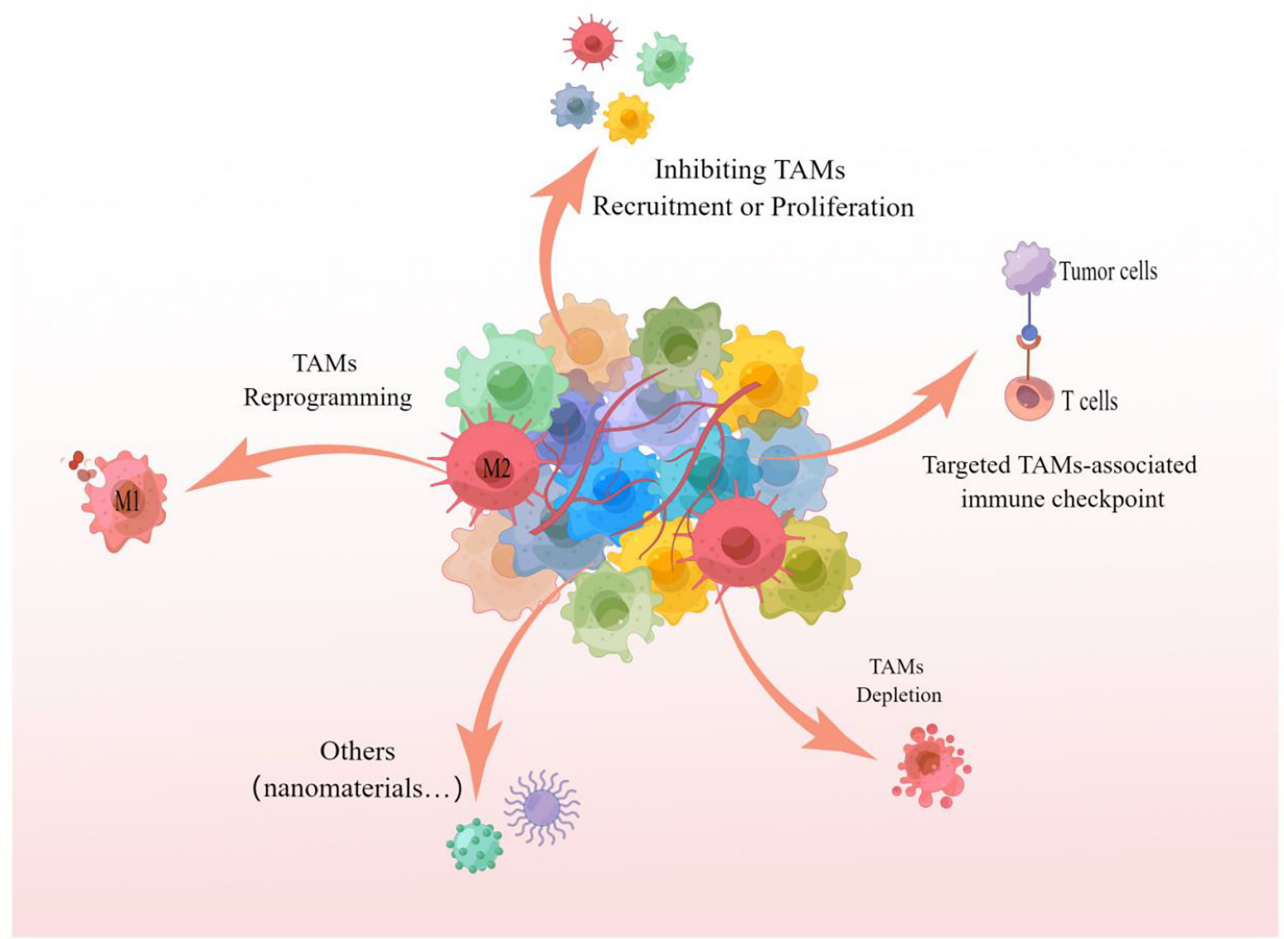
TAMs targeted treatment strategies The picture showed that inhibition of recruitment or proliferation of TAMs, depletion of TAMs, reprogramming of TAMs and targeting of TAMs-associated immune checkpoints are all capable of targeting TAMs for effective anti-tumor therapy. These strategies can likewise be referred to for targeting TAMs for the treatment of pulmonary metastatic melanoma.

As a key component of TEM and/or TMEM, TAMs infiltration is closely related to the survival rate and poor prognosis of tumor patients, and targeting TAMs is becoming an attractive tumor therapeutic intervention strategy (19, 60, 110). Multiple studies have demonstrated that inhibiting the recruitment or proliferation of TAMs, TAMs depletion, TAMs reprogramming, and targeting TAMs-related immune checkpoints are all effective strategies to target TAMs for tumor therapy, the specific treatment methods are shown in **Figure 4**.

### Inhibiting the recruitment or proliferation of TAMs

The recruitment or proliferation of TAMs, which cause the number of TAMs to increase, is a key link in promoting the occurrence, development, and metastasis of tumors. Inhibiting the recruitment or proliferation of monocyte-derived TAMs is considered to be an effective anti-tumor therapeutic strategy targeting TAMs.

Targeting the soluble mediators CSF-1/CSF-1R and CCL2/CCR2, which induce recruitment of TAMs as described above, are currently the main potential targets to inhibit recruitment of TAMs for antitumor effects (111). CSF-1 (CSF-1) interacts with its ligand CSF-1R to promote tumour progression and metastasis by participating in the phenotypic differentiation of M2-type macrophages. Anti-CSF-1R antibodies and CSF-1R inhibitors retard tumour progression by regulating the polarization of TAMs (112, 113). Currently, CSF-1R inhibitors such as PLX3397 and ARRY-382 and anti-CSF-1R antibodies FPA008 and RG7155 are under clinical development or evaluation (114–116). In the mouse xenograft model, the combination of CSF-R inhibitor and anti-PD-1 antibody showed a good therapeutic effect on melanoma (117).

Targeting the CCL2/CCR2 axis exhibits antitumor therapeutic effects by inhibiting the recruitment and polarization of TAMs. The anti-CCL2 antibody Carlumab (CNTO888) can cause a temporary decrease in the level of CCL2 in patients with prostate cancer so that the patient’s disease state can be stabilized (118); CCR2 inhibitor PF-04136309) Combined with FOLFIRINOX (Oxaliplatin + Irinotecan + Leucovorin + Fluorouracil), the clinical outcome of patients with pancreatic adenocarcinoma was significantly improved (18). However, there may also be side effects, when CCR2 inhibitors are combined with chemotherapy or another therapeutic method (119). In a mouse melanoma model, combined treatment of CCR2 inhibitor RS504393 and anti-PD-1 improved the efficacy of melanoma lung metastases in mice (120).

In addition, targeting the CD40 receptor, targeting CX3CL1/CX3CR1, etc. have also been revealed to exert anti-tumor effects by effectively inhibiting the recruitment or proliferation of TAMs (111, 121, 122).CD40, which is expressed on antigen-presenting cells such as dendritic cells, is a member of the TNF receptor superfamily. CD40 plays an anti-tumor effect by promoting the activation of anti-tumor T cells and the polarization of M1 phenotype cells (123). Usually CD40 agonists are used in combination with anti-CSF-1R antibodies to enhance anti-tumour responses by inducing an increase in pro-inflammatory macrophages and eliminating the effects of populations that cause suppressive immune responses (124). For example, a phase I trial of the CD40 agonist APX005M (sotigalimab) and cabiralizumab in combination or not with the PD-1/PD-L1 inhibitor nivolumab for the treatment of anti-PD-1/PD-L1 resistant melanoma, kidney cancer and non-small cell lung cancer (125). There are also multiple clinical trials underway with anti-CD40 antibodies and recombinant CD40 ligands, alone or in combination with other treatments. Such as SGN-40, SEA-CD40, ADC-1013, etc (126).

Overall, Antibodies or small molecules targeting TAMs, alone or in combination with other therapeutic modalities, to inhibit the recruitment or proliferation of TAMs have proven to be a promising therapeutic technique for the treatment of solid tumors including melanoma lung metastases. However, inhibiting the recruitment or proliferation of TAMs also has side effects, so other strategies to target TAMs are also in full swing.

### Depletion of TAMs

Since TAMs are involved in different stages of cancer development and progression, reducing or depleting TAMs is an attractive cancer treatment strategy. Elevated TAM numbers in TEM are often associated with poor prognosis in melanoma patients, therefore, reducing or depleting TAMs can be an effective targeted therapy for melanoma (127, 128).

Numerous of antitumor drugs have cytotoxic effects on TAMs while killing tumor cells (31, 129). Non-cytotoxic doses of commonly used chemotherapeutic drug paclitaxel have been reported to have inhibitory effects on immunosuppressive macrophages in mouse melanoma models, reducing bone marrow-derived suppressor cells (MDSCs), and even blocking the immunosuppressive potential of MDSCs (130). Due to the short biological half-life and renal toxicity of the drug itself, the direct use of TAMs cytotoxic drugs is limited. Combining TAMs cytotoxic drugs with nanomaterials can effectively improve the above problems (131–134). For example, novel clodronate-containing liposomes significantly reduced the number of lung nodules in a B16/F10 lung metastatic melanoma model; This study demonstrated that deletion of TAMs exhibited antitumor effects in metastatic melanoma by inhibiting angiogenesis and regulating inflammation-related cytokines (127). In addition, inhibition of M-CSF receptors can also deplete TAMs and enhance antitumor therapeutic effects (135).

In general terms, TAMs depletion therapy holds great potential as novel cancer (including pulmonary metastatic melanoma) treatment. However, the therapy has also caused a reduction in the number of systemic macrophages, which are the first line of defense for the innate immune response, which can cause adverse effects on the organism (131, 132, 136). Nanomaterials that reduce or deplete TAMs could largely limit the adverse effects of this therapeutic approach on the organism, and as such, much research is underway to target TAMs depleting nanomaterials, which also promises to provide better options for treating lung metastases from melanoma.

### TAMs reprogramming

Due to the plasticity of macrophages themselves and the functional characteristics of TAMs more similar to M2 (pro-tumorigenic) macrophages rather than to M1 (anti-tumorigenic) macrophages, reprogramming M2-like macrophages into M1-like macrophages (also known as “repolarization”) is one of the most attractive antitumor therapeutic modalities for targeting TAMs.

Activation of CD206 (mannose receptor) and toll-like receptors (TLRs) plays an important role in the repolarization of M2 macrophages to M1 macrophages. Studies have shown that activation of CD206 promotes the conversion of M2 to M1 through endocytosis, phagosome-lysosome formation, and autophagy, and enhances the phagocytosis of tumor cells (137). Moreover, studies have shown that the U.S. Food and Drug Administration (FDA)-approved clinical TLR agonist TLR agonist Imiquimod has shown significant antitumor activity in preclinical models of melanoma (138, 139). Another study revealed that the FAD-approved cancer-common chemotherapy drugs sorafenib and paclitaxel can cause repolarization of TAMs (140, 141). Furthermore, if NF-kB inhibition, TAMs differentiate into M2 macrophages, and upon NF-kB activation, TAMs can be redirected to an M1-like phenotype with tumoricidal activity. Studies suggest that immunomodulators such as type I IFN (IFN-α, IFN-β) and type II IFN (IFN-γ) are effective in repolarizing TAMs in skin tumors such as melanoma (142, 143). It has been reported that the M2-specific clearance receptor MARCO is associated with poor prognosis in malignant tumors such as non-cellular lung cancer and metastatic melanoma, suggesting that targeting MARCO may promote the repolarization of M2 macrophages to M1 macrophages (144).

Overall, reprogramming of TAMs is showing promise as a novel anti-tumor targeted therapeutic strategy for advanced or metastatic tumors including melanoma lung metastases. However, clinical data on the therapeutic effect of repolarizers are limited, and more strong evidence is needed to support the efficacy and safety of repolarizers.

### Targeting TAMs−associated immune checkpoints

Immune checkpoint inhibitors (ICIs) are a promising tumor immunotherapy approach for a variety of advanced/metastatic solid tumors, including melanoma, and are now widely accepted by clinicians (145, 146). Numbers of literature show that TAMs involvement in the failure of anti-tumor immune surveillance, as well as the failure of ICIs immunotherapy (146), suggests that targeting TAMs-related immune checkpoints can provide more options for tumor immunotherapy.

Immune checkpoint molecules such as programmed cell death 1 (PD-1), programmed cell death protein ligand 1 (PD-L1) and cytotoxic T lymphocyte-associated protein 4 (CTLA-4) are the most common immune checkpoints in clinical practice’s target. Anti-PD-1/PDL-1 or anti-CTLA-4 therapy mainly maintains an effective immune system against cancer cells by activating tumor-specific cytotoxic T cells (147, 148). One study found that TAMs expressed PD-1 in both mouse and human tumor models; the phagocytic ability of TAMs in a mouse tumor model was negatively correlated with PD-1 expression. When PD-1/PD-L1 expression was inhibited, the phagocytic ability of TAMs *in vivo* was increased, tumor growth was delayed, and mouse survival was prolonged (149). In addition, studies have shown that a specific metabolic enzyme, indoleamine 2,3-dioxygenase (IDO), is involved in T cell exhaustion, which can serve as a target to avoid TAMs-mediated immune escape and improve anti-ICIs efficacy. The combination of IDO and pembrolizumab has good efficacy in the treatment of advanced and metastatic melanoma, and it is worthy of further evaluation (150). A completed Phase 1/2 clinical trial (NCT02073123) of the IDO inhibitor indoximod in combination with ICIs (ipilimumab, pembrolizumab, and nivolumab) in adult patients with metastatic stage III/IV melanoma, the results of the study showed that although the combined treatment method had side effects of fatigue, nausea, and itching, the patients showed good tolerability and efficacy.

Taken together, targeting TAMs-related immune checkpoints has attracted much attention as one of the options for tumor immunotherapy or adjuvant therapy. Regarding the targeting of immune checkpoints related to TAMs, there are not only many mechanism studies but also several clinical trials underway. Immunotherapy is still the common clinical treatment modality for melanoma, especially for advanced and metastatic melanoma. Can targeted TMAs therapy improve the efficacy of conventional immunotherapy? This is undoubtedly worthy of investigation and anticipation.

### Other TAMs targeted therapy strategies and pulmonary metastatic melanoma

Targeted anti-tumor therapy for TAMs in addition to the above-mentioned methods, for example, because TAMs play a key role in angiogenesis and phagocytosis in the process of tumor development and metastasis, there are also corresponding targeted therapy methods (122).

Studies suggested that milk fat globule epidermal growth factor 8 (MFG-E8) promotes melanoma growth by stimulating mesenchymal stromal cell-induced angiogenesis and the differentiation of TAMs to the M2 phenotype, so targeting MFG-E8 to inhibit melanoma angiogenesis may be one of the effective targets for anti-melanoma therapy (151). Monocyte chemoattractant protein (MCP)-1, a chemokine, is one of the key agonists for attracting macrophages to tumors, and a study shows that in melanoma xenografts, inhibition of MCP-1 can inhibit TAMs recruitment and anti-angiogenesis, which is a highly anticipated melanoma therapeutic target (47). In addition, studies revealed a new mechanism by which TAMs promote angiogenesis and melanoma growth through derived adrenomedullin (ADM), which is expected to provide a potential target for melanoma therapy (80).

TAMs have a good phagocytic function for nanomaterials. Studies show that some nanomaterials can induce the intrinsic activity of macrophage phenotype differentiation so that TAMs can repolarize to the M1 phenotype to achieve the purpose of anti-tumor therapy (152, 153). For example, a hyaluronic acid-coated, mannan-conjugated MnO2 nanoparticle (Man-HA-MnO2 NPs) repolarized TAMs to the M1 phenotype, alleviated tumor hypoxia by significantly enhancing tumor oxygenation, and thus Anti-tumor therapeutic effect; and this nanomaterial combined with doxorubicin can synergistically inhibit the growth and proliferation of tumor cells (154). Nanomaterials can also resist the progression of melanoma by anti-tumor angiogenesis and other means (94, 154, 155).

The above studies suggest that targeting TAMs therapy is an attractive and potential cancer treatment strategy. However, the specific choice of a treatment strategy for melanoma lung metastases or other different types of tumors should be based on the actual situation of the tumor and the role of targeted TAMs.

## Conclusion and perspective

It is generally believed that macrophages play an important role in the development and metastasis of various solid tumors, including melanoma. Early dissemination and late multi-organ metastasis colonization are typical features of melanoma. Among the multiple organs that skin melanoma may colonize, the lung is one of the most common distant metastatic sites of melanoma. Lung metastases from melanoma account for 5% of all malignant metastases and have a high lethality rate. The macrophages involved in the lung metastasis of melanoma can refer to the macrophages in the occurrence and development of other tumors, and their sources may be monocyte-macrophages or tissue sources. Generally divided into two types: M1 macrophages and M2 macrophages. M1 has pro-inflammatory and anti-tumor properties, while M2 has anti-inflammatory and pro-tumor properties. TAMs are considered to belong to the M2 phenotype because their functions in promoting tumorigenesis, tumor angiogenesis, and metastasis are more similar to M2 macrophages.

Several studies have revealed the effect of TME on tumorigenesis and development and the effect of TMEM on tumor metastasis. TAMs, as the main component of the TME and TMEM, is a complex heterogeneous population of cells that contribute to the malignant features of solid tumors. This paper summarizes the role of TAMs in promoting tumor cell invasion and in participating in various steps of the tumor metastasis cascade, and the ways in which regulation such as epigenetics affects the function of TAMs and thus tumor progression and metastasis, which may provide some explanation for the role of TAMs in melanoma lung metastasis. In addition, the heterogeneity and specificity of TAMs lay the foundation for the development of therapeutic approaches targeting TAMs. In this paper, we also summarized anti-tumor therapeutic approaches targeting TAMs such as, inhibiting the recruitment and proliferation of TAMs, depleting TAMs, repro- gramming TAMs and targeting angiogenesis. It is also worth noting that in solid tumors including melanoma, both basic and clinical trials on TAMs are in full swing, suggesting that targeting TAMs strategies are expected to form a precise treatment and will be a valuable anti-tumor treatment strategy in the future.

In conclusion, TAMs have diverse functions in TME and TMEM and play complex roles in various solid tumors. Studying the role of TAMs in the process of melanoma pulmonary metastasis and the therapeutic strategies targeting TAMs is expected to provide more possible mechanism explanations and treatment options for melanoma pulmonary metastasis.

## Author contributions

KX and SC drafted an outline of the manuscript. KX drafted the abstract, generated the figure, and edited the manuscript. MQ drafted the discussion and edited the manuscript. TS edited the manuscript. JZ added to and edited the manuscript. All authors contributed to the article and approved the submitted version.

## Funding

This work was supported by the National Natural Science Foundation of China (under Grants 82073018, and 82073019) and also funded by the Shenzhen Science and Technology Innovation Committee (JCYJ20210324114212035).

## Conflict of interest

The authors declare that the research was conducted in the absence of any commercial or financial relationships that could be construed as a potential conflict of interest.

## Publisher’s note

All claims expressed in this article are solely those of the authors and do not necessarily represent those of their affiliated organizations, or those of the publisher, the editors and the reviewers. Any product that may be evaluated in this article, or claim that may be made by its manufacturer, is not guaranteed or endorsed by the publisher.
